# The Sponge-Tent in Its Practical Application

**Published:** 1884-01

**Authors:** Marcell Hartwig

**Affiliations:** Buffalo, N. Y.


### BUFFALO

### Medical and Surgical Journal.

VOL. XXIII. JANUARY, 1884. No. 6.

### Original Communications.

### THE SPONGE-TENT IN ITS PRACTICAL APPLICATION.*

BY MARCELL HARTWIG, M. D., Buffalo, N. Y.

The subject may seem stale, for it is forty years since Dr. Simpson first introduced the sponge-tent among the most important of our resources for diagnosis. Nevertheless, I consider the sponge-tent one of the most important inventions of practical medicine. Its use in utero is certainly most valuable; but for other purposes, likewise, it can be employed most advantageously, and with eminent success. Thus, as you know, Dr. Bartlett has used the tent successfully to stop bleeding from the nose. Probably most of his cases were dependent upon a relaxation of the tissue of the Schneiderian membrane; and by long-continued pressure the mucus membrane here, just as in the uterine cavity, becomes considerably changed in its nutrition, and in consequence thereof ceases to bleed. Another purpose which the sponge-tent admirably answers, is the dilatation of fistulæ, or to irritate them, so as to excite granulation, by annointing the tent with irritating salves. I have read of cases of fistulæ in ano which were cured in this manner. We shall

* Read before the Buffalo Medical and Surgical Association, Tuesday evening, Dec. 4, 1883.

mention other uses for the tent later on, but at present let us consider its principal application, its employment in the uterine cavity.

All important with the use of the tent here, as elsewhere, is strict antisepsis. This knowledge is not wanting; but the practical treatment of the sponges, as we find them in the market, is not at all what is desirable. The market offers (boastfully as always) carbolized, disinfected, compressed sponge-tents, without considering that carbolic acid is volatile, and that the surface, at all events, remains without disinfection, the less so because the buyers generally handle a great many of the tents, while they purchase a few only, until after a while the whole stock has been handled and looks grayish-black, so that one feels almost nauseous when he looks at them. Another kind is coated with wax or paraffine, and looks decidedly black. If both sponge and coating were carefully bleached, the appearance would, of course, be far more inviting. Two other objections attach themselves to the tents. At present the market offers for sale the finest sizes only in a bent shape, but though bent uteri generally need larger sizes, these cannot be had, the larger sizes being made in straight shape. Furthermore, most of the tents are made of the coarsest kind of sponge, and thus they possess a great deal of expansibility, but no resistance. Accordingly, they do not expand to more than one-fourth of their size when the uterus is at all rigid; the finest and most compact sponges would appear to be precisely good enough. Our instrument makers should take the hint. A third objection, the possibility to tear out the thread fastened to the sponge, has been ingeniously overcome by our lamented James P. White, by running a loop of wire, or piece of string, through its entire length. But such an occurrence, of tearing of the thread, may be avoided if the sponge is of good quality, and if we take sufficient time in withdrawing it, by pulling gently but at the same time very steadily for several minutes. Still, it is better to be on the safe side always, and hence to use White's tents.

But all these objections would lose much of their force, were the disinfection of the sponges thorough and reliable. In future, non-evaporating substances will have to be used, such as salicylic acid, five per cent.; iodoform, five per cent.; or bichloride of mercury, one-half per cent.

According to recent investigations, that, namely, the internal surface of the uterus, is always teeming with bacilli, we should always apply, even though the tent is well disinfected, a reliable antisepticum to its surface, in order not to press *living* bacilli into the tissue of the uterus itself. For this purpose I recommend dipping the tent into a ten per cent. solution of carbolized glycerine and then to roll it in powdered iodoform. The combination offers the advantage that it does not soften the sponge; and as to the glycerine, it has been shown to possess a valuable property in that it produces a profuse, watery secretion from the uterine cavity, and that it thus manifests a depletory action. This was pointed out and proven by the late Marion Sims, long the pride and founder of American gynecology. The iodoform has, on account of its slight solubility, the advantage of continuing antiseptic action for a greater length of time. To be sure, the introduction removes a great deal of the antiseptic, but *semper a liquid hæret.* Just on this account the sponge ought to be not only disinfected, but ought to contain a free disinfecting agent, ready for action. If the sponge is coated with wax or paraffine (gelatine, perhaps, is preferable) the coating, also, should contain an antisepticum. Sponges ought to be coated, because with a narrow uterine canal, such in which dilatation is so frequently necessary, the introduction is often not immediately possible, and the sponge is spoiled before introduced, unless it is coated. For this reason I generally begin with tupelo tents, since their introduction. They, too, should be disinfected, though they are not so liable to infect, and certainly never did in my hands. It is to be regretted that no bent tupelo can be had, and Tieman & Co. have recently informed me that they have not succeeded in making any as yet. Let us hope that ere long they may succeed. But till then our own dexterity must help us to introduce a straight tent into a bent womb.

Another method of introducing tents is a preliminary, limited dilatation with steel instruments. But for a complete dilatation they all are unfit, according to my conviction; and were they not, this proceeding itself would be altogether reprehensible, being too rude. I have seen a nervous lady faint with a shriek from a free and easy introduction of a sound, when the button passed the inner mouth of the womb. I convinced myself twice, on this same lady, *experimenti causa* to a certain extent, of this intense sensitiveness of the internal os, which is always peculiarly irritable.

After a thin tupelo has been laying in utero for six hours, we can generally succeed to introduce a little thicker sponge-tent. It is always advisable to wash the uterine cavity with a three per cent. solution of carbolic acid, before a new tent is introduced. If a very wide dilatation is necessary, several sponges must finally be introduced at the same time. As a rule, a sponge ought not to be left in the uterus more than twelve hours, and all instruments, and the vagina, should be thoroughly disinfected. It was at one time proposed to disinfect the uterus at the very outset by washing its cavity, but this is not always possible, because the fluid cannot safely escape, even by suction. Many grave accidents have thus happened. Reversely, to be able to apply medicines to the uterine cavity, previous dilatation is often necessary.

### INDICATIONS FOR THE USE OF THE SPONGE-TENTS, AND MY PERSONAL EXPERIENCE.

Time does not permit, on this occasion, to be exhaustive, or even to consider the literature of the subject; but I believe that I have made use of the sponge-tent in a sufficient number of cases to warrant giving my own observations, which may not be without interest. I shall speak especially of my unlucky cases, because they are at all times far more instructive, though, unfortunately, so often suppressed.

The employment of the sponge-tent in gynecology may be divided, with more or less propriety, into its use as an explorative measure and into its use as a curative agent. As a means of curing, the tent is useful in destroying, by its continued pressure, the granular degeneration of the mucous membrane. It may be possible to cure flexions occasionally by means of the tent alone, but I do not know of such a case, though the straightening, in combination with the change of nutrition, looks promising. For the latter purpose alone, we occasionally use the tent in the chronic infarctus, and with more or less success. To destroy granulations and the concomitant leucorrhœa once proved grandly efficient in my hands. A lady, who had inherited from her mother a dysmenorrhœa membranacea, and who suffered since her nuptials from a very profuse (perhaps Noeggerath's) leucorrhœa, commenced to flow considerably during her menses, while the usual pains were still increasing. The leucorrhœa having persisted during four gestations, the everted mucus membrane of the os being highly reddened, pulpy and bleeding at the slightest touch, I supposed there were granulations on the inner surface, and proceeded with dilatation. Three tents were introduced into the uterus and allowed to remain twenty-four hours. The reflex and other phenomena were intense. Very often repeated vomiting and strong pains were only incompletely controlled by subcutaneous injections of morphia, but after removal of the last sponge the leucorrhœa of twenty years' standing was forever gone, and the menses became regular and painless. After three months some membranous pieces began, with some pain, to show again; but the leucorrhœa did not come again for years. Was my diagnosis of granulations correct or not? Who will deny it, or who could prove it beyond doubt?

In two other cases of dysmenorrhœa membranacea the effect was similar, but not as perfect; the leucorrhœa continued, to a certain extent, but I did not succeed, in one of these cases, to introduce a second tent to the fundus, and had to stop.

In regard to straightening a bent uterus with the tent, I would add that certainly few cases would be adapted for it. When adhesions fasten the womb or where one wall is atrophied at the inner mouth, the tent must of necessity fail. But in cases of retroflexions from softness of the uterine tissue *post partum*, a tent, and lying on the abdomen, may show results yet.

With chronic uterine infarctus I have used the sponge twice. In both cases there existed laceratio ossis externi and enlargement of the womb by one-half of an inch, but they differed, as the second case was a plethoric lady with excessive menses, while the first was more delicate and had rather scanty monthlies. The initial result was satisfactory in both cases. The leucorrhœa diminished in both, but in both nothing was finally achieved. The result is the same as Fritsch gives in the *Ctbl. f. Gynecologie*, p. 28, 1883. Nevertherless, I entertain hopes that definite results will follow in some cases, because the nutritive changes, through the extensive dilatation, must be considerable. Absorption of the inflammatory thickening of the uterine wall ought to be excited, but practical experience, not theoretical considerations, will have to decide.

One momentum will have to be kept in view, a pathologico-anatomical and etiological diagnosis, not a mechanical one, as has been inaugurated by Sims' influence through his magnificent gift to the profession—his uterine surgery.

The second of the above-mentioned cases is not entirely cured yet, and perhaps never will be; but the interest of the case centres in a disagreeable change it assumed. The introduction of the first *bent* sponge was comparatively easy, though considerable anteflexion existed; but the second *straight* sponge could not be entirely introduced, as in a case already mentioned, though I omitted to wash the uterine cavity purposely, in order not to give the uterus time to contract. Whether on this or any other account, and though I had used all the disinfecting precautions mentioned above, the fact is, that forty-eight hours after the first introduction a fever set in. For three days it fluctuated between 101 and 103½, then it sank to 100, to 101, and continued, though the lady left the bed, and though she did not present any untoward symptoms beyond a little weakness and some of her old complaints, for three weeks. The closest examination did not reveal anything like para- or perimetritis, or the like. Where have we to place the cause of this fever? I cannot but think that somewhere the disinfecting precautions were at fault anyhow, but that the infection was so slight as to cause at least no perceptible parametritis, etc.

A very instructive case in regard to infection, in which to my great sorrow, no post mortem examination could be obtained, was that of a robust woman whose sterility I tried to cure by slitting the portio vaginalis, using as a dressing to the wound per chloride of iron and cotton dipped in glycerine, as Sims recommended.

The treatment was carried on at my office. On the fifth day I took it for granted that the wound would be in a condition non-susceptible to septic poison, because it was granulating; and hence removed the tampon. Already, on the next day, she became feverish, but I was summoned one day later. I found her with considerable fever, disinfected the uterine cavity and wound; but it nevertheless took three weeks until the fever subsided altogether. Here likewise it was impossible to find a local inflammation. She had been up and about her duties again and seemed to be well and cured of her trouble, when once more a fever began to set in. By and by, a chronic (apparently cheesy) pneumonia was diagnosed in right lower lobe, which, at the end of about two years, caused her death. Her womb had not troubled her any, and at the beginning of her pneumonic symptoms I was thinking of embolism. May be that she had inherited a taint from her father, whose history, however, is not known, or that her constitution was undermined by indulgence in strong drinks; still, nobody would have suspected phthisis in her, and I could not help thinking that in this instance there was something anyhow in the *post hoc ergo propter hoc*, the more so, because a similar state of things occurred again to me, the development, namely, of cheesy pneumonia shortly after I had dilated a cervix with metallic dilators. I once saw pyæmia with embolism in the lungs after abortion, resembling in some respects these cases, although, of course, *toto coelo*, different in its pathogenesis. This case recovered. I would remark, in passing, that among my cases in which I incised the portio, there was one which commenced with a fever from the same cause as the above mentioned fatal one. But being called at the very beginning, I immediately suppressed the fever by means of thorough disinfection.

The question about uterine operations in connection with phthisis deserves closer study, all due respect being had for Koch's investigations.

Far oftener than for curative purposes, the tent is used as a means of diagnosis, and to enable us to operate in the inside of the uterus in non-pregnant and pregnant women, and after timely or untimely confinements. Here the tent often wins its best triumphs. Frequently we would be entirely unable to do anything really beneficial for our patients had we not the tent at our disposal. But the danger of infection should at all times be kept in mind. Do not use it when you can get at a diagnosis with simpler means, or where the disease is not of much importance; but if you do use it, do not omit any of the indicated precautions, whether they appear practical or theoretical. No thought can be more horrifying to the physician than to know positively that he has injured instead of done good, or where with due care he could have saved. And just here an insignificant negligence can make you pay dearly. My first case, in Berlin in 1873, has taught me a severe lesson. A lady, in the climacteric period, called on me, having a protracted menstruation, which lasted two months uninterruptedly. Being desirous of finding the cause, other means having failed to detect it, I inserted one of those extremely rudely prepared tents then kept by our instrument dealers. They were, as far as my recollection goes, supplied with antiseptic precautions, but were without complete antiseptics. That the latter was incomplete was best proven by the effect. The lady became very sick and dismissed me. I afterwards learned from a colleague that she recovered after a parametritis of six or more weeks' duration. For a long time the recollection of the case caused my head to ache; for a long time the woman's pale, haggard face and reproachful eyes disturbed my nights' rest.

For a long time I abstained altogether from using the tent; but in the course of time, necessity forced me to employ it again.

One year later, in the country, I was called to see a woman who bled profusely post abortum. Ice in the vagina, vaginal medicated injections (hot injections were then unknown), ergotin, iron-chloride tampon, all failed, helping a short time only at best. My fear of seeing her bleed to death was greater than it would be to-day. I went back to the tent, my antisepsis was more stringent. Pasteur's and Lister's results had penetrated Germany deeper. I dilated, scraped with the sharp spoon, and the hemorrhage was at an end. Later on, I had a very similar case, in which I know that I did not forget to finish with a thorough syringing out of the womb with a four per cent. carbolic acid solution. In this case, the result was so perfect that a year afterward I had the pleasure of delivering the lady of a full-grown child. In cases in which the mouth of the womb is not patulous enough to allow of scraping it without previous dilatation, Fritsch recommends to scrape with an "ear-spoon," so as to avoid the tent. While recognizing the authority of Fritsch, I have my doubts whether cases, which require scraping, would yield *complete* results thereby. Besides, the diagnosis remains obscurer, as you cannot, as a matter of course, introduce the finger into the womb. Of late years, I have not had much opportunity of forcible treatment of those cases of abortion, because the more I saw, the more I was convinced that a great deal of loss of blood could be borne innocuously, and thus I treated most of those cases where I would use forcible means formerly, on the expectant plan merely. Nature's efforts succeed remarkably well with the abortus as well as with retained placentæ, in which the loss of blood would seem to lead to death, before nature has finished her work.

In regard to the cases of retained placentæ after abortion, two essays recently appeared by Munde and Sweringen, which oppose each other diametrically. While Munde urges active proceedings, basing his argument on fifty cases, Sweringen rejects this advice *in toto*, and considers the expectant treatment the only proper course to pursue. Here, as elsewhere, the good lies in the *aurea mediocritas*, in the golden middle course; but where this course is, is by no means easy to say. Only a large experience and much study will teach us to find it. Probably, the most difficult decision lies with cases where sepsis of the placenta is already ushered in. For the most part, the mouth of the womb will be patulous enough for immediate scraping and disinfection, and that, according to my idea, would be the most proper proceeding. But what is to be done when peritonitis generalis is present already? "*Noli me tangere*," may be the correct rule in most cases, although I saw such a case end fatally with "don't touch me." It would be impossible to exert pressure upon the abdomen in such cases to steady the womb; for a woman who has general peritonitis cannot stand much pressure; but dilatation and scraping may sometimes be possible, because the peritonitis varies in intensity in different cases, and we can hold the uterus sufficiently steady per vaginam. For active proceeding, we can here adduce the general experience that the new introduction of septic poison into the blood ceases with the washing out of the womb, and that instead the antiseptic is absorbed, although, to be sure, not too energetically, after abortions occurring during the first three months. Thus we close the *pons et origo* of the sickness, while the septic substances already in the circulation may yet be destroyed by the oxidizing power of the organism. After timely confinements in which no scraping was necessary, I have cured three cases of general peritonitis by means of this disinfecting process. Why should it not be possible after abortions? You will certainly have to see your patient early would you see her survive.

If, in such a case, the mouth of the womb should be closed, and the sponge-tent necessary to dilate ere you can, of course, scrape and disinfect the inside of the womb, what then?

I have, as yet, not had such a case, and should like to hear of one from our members, although it is rather improbable that such a case should occur, inasmuch as the septic process generally commences within three days post abortum, and keeps the uterus relaxed. At any rate, the sponge here would have easy work; nor would it need to lay long in the uterus. The effect of the sponge's squeezing septic substance into the wall of the womb would be compensated by the antisepticum applied to its surface.

I once had an ante-abortum case which ran a very bad course. Despite the fact that I had several cases in which I used the sponge with good results, some of those cases in which abortion was designedly induced, still I believe that there are better means than the sponge for this purpose. In the case just mentioned I yielded somewhat to the pressure brought to bear on me to hasten the abortion, though against my convictions, because I have become more and more convinced of the fact that the slower an abortion takes place the better will it be; the less likelihood of retained placentæ. We should not, however, lay aside our convictions to any amount of earnest appeals to proceed actively and hurriedly, unless we see indications of imminent danger from flooding, or unless the thermometer indicates a rise of temperature. In this case the sponge was expelled with the ovum. Having removed everything, and while about to syringe out the womb, I was called away in great haste. The consequence of this neglect was that after five days (probably so late because the sponge was well disinfected) fever manifested itself. Disinfection of the womb, quinine, etc., caused its disappearance in three weeks. There was not a very well defined parametritic tumor on the left side.

In cases of flooding from intra-uterine polypi, the tent has achieved its greatest triumphs. In such a case it actually saved the life of a woman who had bled nearly the year round. Her medical attendant was extremely despairing of the case as he requested me to see it in consultation. Active dilatation with three sponges, and forcible scraping out of several large and many small polypi, together with Routh's injection, gave the dying woman a new lease on life, and she still lives. But it took two months before she could go round about again, though no fever was present. At the time of the operation her pulse was wiry, her color like a sheet of paper, and her legs œdematous.

(I will give, in parenthesis, the formula of Routh's injection, and remark that it is far preferable to solutions of iron for purposes of injections into the womb, because it does not form any coagula, though it has great styptic properties. This is the formula:

| | | |
|---|---|---|
| ℞ | Iodinii, - - - - | ʒi |
| | Potasii iodidi, - - - - | ʒii |
| | Alcoholis, - - - - | ℥ii |
| | Aquæ, - - - - - | ℥vi). |

It is known that with the aid of the tent intra-mural polypi can often be enucleated. Thus far, no such case has occurred to me.

Let me briefly mention yet a few other indications in which I would use the sponge-tent. I take the liberty to propose its use for dilating the œsophagus. The treatment must be gentler than when sounds are used. I would take a tupelo or gelatine-coated tent, tapering to both ends, and fastened to a long, strong thread. Draw the thread through an œsophageal sound, the rounded end of which is cut off. With the thread in tension, the tent would stay partly in the tube, and so could be guided with it. Having gotten it well between the constricting parts, withdraw the sound and leave the thread hang out of the patient's mouth.

In a similar manner strictures of the urethra and of the rectum could perhaps be dilated, and with greater advantage than by the methods now in vogue.

Since writing this, Prof. Bergmann has dilated with the tent an œsophageal stricture from the stomach side, after gastrostomy.

P. S.—In the discussion of this paper by the society, it was mentioned that the tupelo-tents ought to be used almost exclusively, just because of the dangers of the sponge-tent, as set forth in this article. The author replied, in summing up, that this was precisely what he meant; the very intention of the paper being to put the medical public once again on their guard in respect to sponge-tents, although he did not mention the many cases in which he used the sponge-tent without any cause for complaint. With tupelos, he did not have, so far, any untoward accidents either, but his experience with them is very moderate yet.

